# Delayed Recognition of Glycoprotein IIb/IIIa Antibody-Associated Platelet Dysfunction in Chronic Liver Disease: A Platelet-Mediated Bleeding-Thrombosis Dysregulation

**DOI:** 10.7759/cureus.112237

**Published:** 2026-07-07

**Authors:** Niyas Khalid Ottu Para, Seema Rab

**Affiliations:** 1 Internal Medicine, Burjeel Hospital, Abu Dhabi, ARE; 2 Internal Medicine, Burjeel Holdings, Abu Dhabi, ARE

**Keywords:** autoimmune bleeding disorder, chronic liver disease, glycoprotein gpiib-iiia, platelet antibodies, thrombosis

## Abstract

Recurrent bleeding in chronic liver disease is frequently attributed to structural gastrointestinal pathology, often without detailed interrogation of platelet function. We report a patient with prolonged transfusion-dependent gastrointestinal bleeding in whom repeated endoscopic findings failed to account for the severity and persistence of hemorrhage.

The clinical course was characterized by a reproducible pattern in which bleeding episodes led to temporary withdrawal of aspirin with partial hematologic stabilization, whereas recurrent cardiac biomarker elevation prompted reintroduction of antiplatelet therapy, precipitating further bleeding. This pattern was consistent with platelet-mediated impairment of primary hemostasis amplified by pharmacologic platelet inhibition.

Platelet-focused evaluation ultimately demonstrated glycoprotein IIb/IIIa antibody reactivity, indicating qualitative platelet dysfunction rather than isolated thrombocytopenia. Recognition of this possible platelet-mediated mechanism reframed the illness as a dynamic hemostatic instability state operating within chronic liver disease.

Permanent withdrawal of aspirin resulted in cessation of overt bleeding, elimination of transfusion requirements, and sustained hematologic stabilization over more than three months of follow-up.

This case illustrates a reproducible clinicophysiologic pattern, referred to here as a platelet-mediated hemostatic instability cycle, in which competing bleeding and thrombotic priorities perpetuate recurrent clinical instability. The report emphasizes the importance of platelet function assessment, longitudinal pattern recognition, and mechanism-based clinical reasoning in complex multisystem disease.

## Introduction

Hemostasis in chronic liver disease is increasingly recognized as a dynamically rebalanced yet inherently unstable system rather than a purely hypocoagulable state. Reductions in platelet number, procoagulant factors, and endogenous anticoagulant pathways coexist with endothelial activation, elevated von Willebrand factor (vWF), altered fibrinolysis, and complex platelet signaling abnormalities, creating a fragile physiologic equilibrium that may manifest clinically as either bleeding or thrombosis depending on prevailing biological and therapeutic influences [1-4].

In routine clinical practice, recurrent gastrointestinal bleeding in cirrhosis is frequently attributed to structural abnormalities such as portal hypertensive gastropathy, hemorrhoidal disease, or mucosal erosions. While such lesions are commonly present, they may not adequately explain cases in which bleeding is severe, recurrent, transfusion-dependent, and refractory to standard interventions. In such settings, disorders of primary hemostasis, particularly platelet dysfunction, should be actively considered [1-4].

Platelet aggregation depends critically on glycoprotein IIb/IIIa (integrin αIIbβ3), the final common pathway for fibrinogen-mediated platelet cross-linking. Disruption at this level can impair clot formation despite relatively preserved conventional coagulation parameters [5,6]. In parallel, recurrent severe anemia imposes substantial hemodynamic stress on the myocardium through impaired oxygen delivery and compensatory increases in cardiac workload, frequently resulting in troponin elevation and escalation of antiplatelet therapy [7-9]. In susceptible patients, this may generate a self-perpetuating cycle in which treatment of perceived thrombotic risk amplifies bleeding vulnerability.

Current European Society of Cardiology (ESC) guidance recommends individualized antiplatelet strategies in patients at high bleeding risk following PCI, including consideration of shortened dual antiplatelet therapy and transition to single antiplatelet therapy when clinically appropriate. However, evidence remains limited in patients with advanced chronic liver disease and concurrent platelet dysfunction, making individualized multidisciplinary decision-making essential.

Although portal hypertensive lesions, peptic ulcer disease, and other structural gastrointestinal lesions account for the majority of bleeding episodes in patients with cirrhosis, a subset of patients experience recurrent bleeding that appears disproportionate to identifiable endoscopic abnormalities. Qualitative platelet dysfunction is common in chronic liver disease due to impaired platelet activation, receptor abnormalities, altered intracellular signaling, and defective interactions with vWF, although its true prevalence remains uncertain because platelet function testing is not routinely performed. Clinically significant immune-mediated platelet dysfunction is considerably rarer and remains poorly characterized. Recognition of these nonstructural contributors may therefore be important in selected patients with recurrent bleeding despite apparently adequate management of structural lesions.

We present a patient in whom these processes interacted in a reproducible longitudinal pattern. The case demonstrates how delayed recognition of platelet-mediated dysfunction resulted in prolonged morbidity and introduces a clinically relevant framework, referred to here as a platelet-mediated hemostatic instability cycle, to explain the observed bleeding-thrombosis dynamics.

## Case presentation

A 60-year-old male with a history of coronary artery disease status post percutaneous coronary intervention, chronic liver disease with Child-Pugh B cirrhosis (score 7), portal hypertensive features, hypertension, chronic obstructive pulmonary disease, and prior alcohol use presented with a prolonged history of recurrent gastrointestinal bleeding.

Over several years, the patient experienced repeated episodes of melena and bleeding per rectum, frequently associated with profound fatigue, exertional dyspnea, palpitations, dizziness, and marked functional limitation. These episodes were severe and repeatedly necessitated hospital admission and packed red blood cell transfusion, with hemoglobin levels recurrently falling to approximately 6-8 g/dL, usually requiring 2-3 units of packed red blood cells (PRBCs). The bleeding phenotype was variable, alternating between overt rectal bleeding and features suggestive of ongoing occult gastrointestinal blood loss.

Multiple endoscopic evaluations were performed during the early and intermediate phases of illness. Upper gastrointestinal endoscopy demonstrated erosive gastritis, while colonoscopy identified hemorrhoidal disease, for which banding procedures were performed. Despite these interventions, the patient continued to experience recurrent bleeding. Subsequent evaluations revealed portal hypertensive gastropathy, mild colitis, and duodenal nodular lesions. Histopathological analysis demonstrated benign findings, including Brunner gland hyperplasia and erosive inflammatory changes without dysplasia or malignancy. Although these abnormalities provided potential bleeding substrates, they did not adequately explain the severity, persistence, or transfusion dependence of hemorrhage.

During the course of his illness, the patient developed non-ST-elevation myocardial infarction (NSTEMI). Following coronary angiography, percutaneous coronary intervention was performed with initiation of dual antiplatelet therapy. From this point onward, a reproducible clinical pattern emerged across multiple admissions. Episodes of gastrointestinal bleeding were frequently accompanied by troponin elevation consistent with type 2 myocardial infarction secondary to profound anemia and myocardial oxygen supply-demand mismatch, prompting cardiology consultation and reinforcement of antiplatelet therapy because of perceived thrombotic risk.

A cyclical pattern became increasingly evident over time. This pattern was reproducible across multiple admissions and became particularly apparent when reviewed longitudinally rather than as isolated clinical events. During bleeding episodes, aspirin was withheld, resulting in reduction of overt bleeding, stabilization of hemoglobin levels following transfusion, and partial recovery of platelet counts. However, because of recurrent troponin elevation and underlying coronary artery disease, aspirin was subsequently reintroduced. Following reintroduction, bleeding recurred, often within a short interval, leading to further hospitalization. This pattern of alternating aspirin withdrawal and reintroduction persisted over an extended period and reflected competing management priorities between bleeding control and cardiovascular risk mitigation.

Laboratory evaluation consistently demonstrated chronic microcytic anemia with elevated red cell distribution width, compatible with iron deficiency secondary to chronic blood loss. Hemoglobin fluctuations were closely associated with transfusion events rather than spontaneous hematologic recovery. Platelet counts remained persistently reduced, generally fluctuating between approximately 65 and 109 × 10⁹/L, with relative improvement observed during periods when aspirin was withheld. Importantly, platelet recovery demonstrated a consistent temporal association with aspirin withdrawal, suggesting a functional component in addition to thrombocytopenia related to chronic liver disease. Importantly, platelet counts did not demonstrate the marked spontaneous fluctuation typically expected in untreated immune thrombocytopenia but instead showed relative longitudinal stability with reproducible improvement after aspirin cessation. Conventional coagulation parameters remained relatively preserved throughout the clinical course.

**Figure 1:**
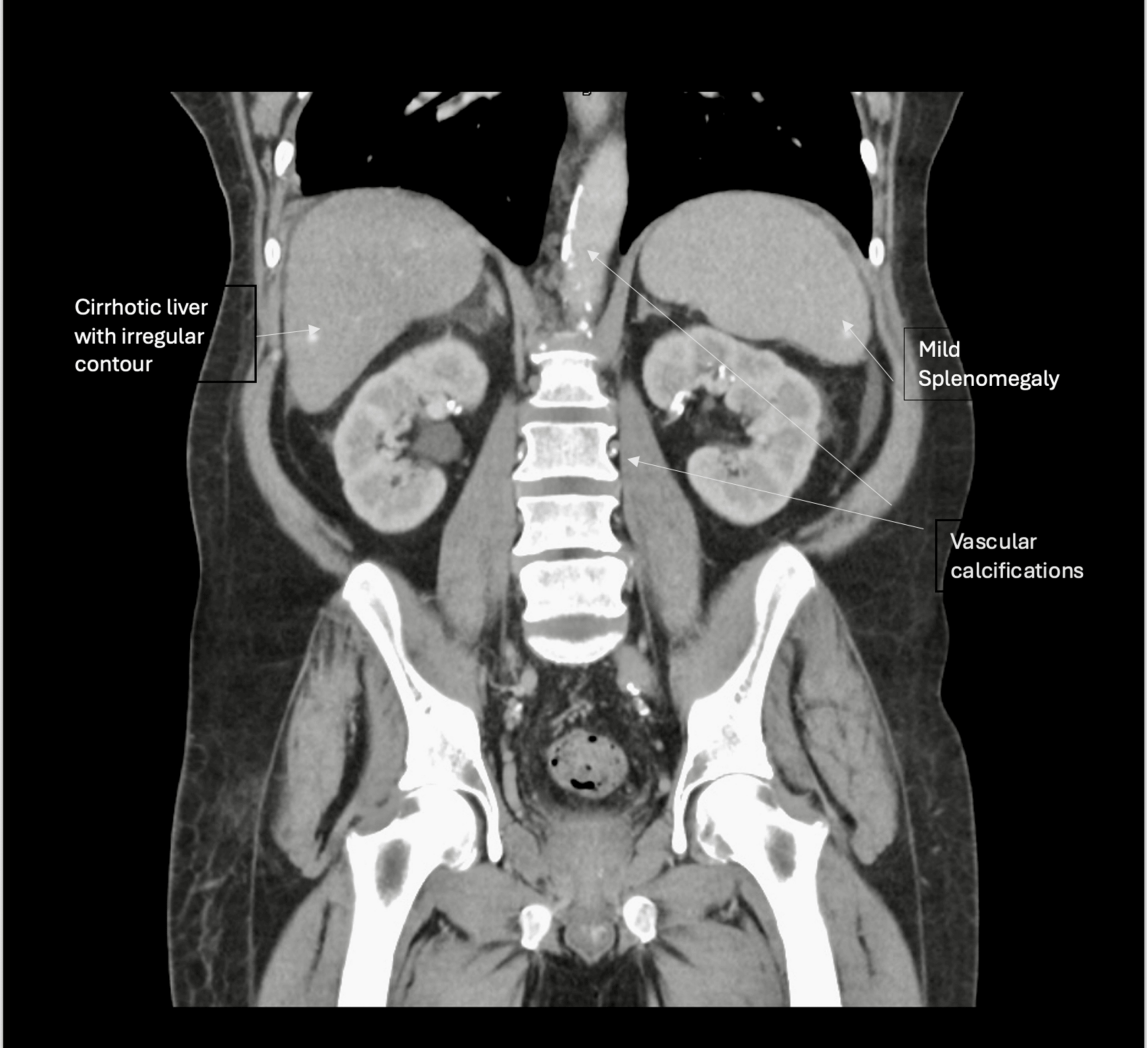
Contrast-enhanced computed tomography of the abdomen demonstrating chronic liver disease and splenomegaly Coronal contrast-enhanced CT image showing hepatomegaly with irregular liver contour, mild splenomegaly, and extensive vascular calcifications involving the abdominal aorta and major abdominal vessels. No portal or splenic vein thrombosis was evident on imaging.

Computed tomography imaging and angiographic studies performed at different time points did not identify a definitive vascular source of bleeding. Echocardiographic evaluation demonstrated mild aortic stenosis without significant systolic dysfunction. A contrast-enhanced CT image showing hepatomegaly with irregular liver contour, mild splenomegaly, and extensive vascular calcifications involving the abdominal aorta and major abdominal vessels is shown in Figure 1.

Given the disproportionate severity of bleeding despite relatively preserved conventional coagulation parameters and the absence of a definitive structural source, an immune-mediated platelet dysfunction was considered, prompting platelet antibody testing. This demonstrated positive platelet glycoprotein IIb/IIIa antibody reactivity. Additional laboratory findings included elevated vWF antigen and activity, together with reduced Protein C and Protein S levels, interpreted cautiously in the context of underlying chronic liver disease. Key laboratory findings supporting platelet dysfunction and hemostatic imbalance are summarized in Table 1.

**Table 1:** Key laboratory findings supporting platelet dysfunction and hemostatic imbalance HLA: Human leukocyte antigen; CLD: Chronic liver disease.

Test	Result	Unit	Reference range	Interpretation/relevance
GPIIb/IIIa antibody	Positive	Qualitative	Negative	Key abnormality; supports platelet receptor-level dysfunction
HLA class I antibody	Negative	Qualitative	Negative	No HLA class I antibody detected
Ia/IIa antibody	Comment	Qualitative	Negative	Broad platelet antibody comment; specific antibody to Ia/IIa antigen could not be ruled out
GPIb/IX antibody	Negative	Qualitative	Negative	No GPIb/IX antibody detected
Glycoprotein IV antibody	Negative	Qualitative	Negative	No GPIV antibody detected
Protein C activity	52	%	70–130	Low; reduced endogenous anticoagulant reserve in CLD
Protein S activity	48	%	77–143	Low; reduced endogenous anticoagulant reserve in CLD
vWF activity	>200.0	%	50–200	Elevated; endothelial activation/compensatory primary hemostasis marker
vWF antigen	344	%	50–160	Markedly elevated
Factor VIII activity	137	%	60–150	Normal-high; prohemostatic/prothrombotic component of rebalanced CLD hemostasis
Vitamin K1	0.34	ng/mL	0.10–2.20	Within reference range; argues against vitamin K deficiency as main driver

**Figure 2:**
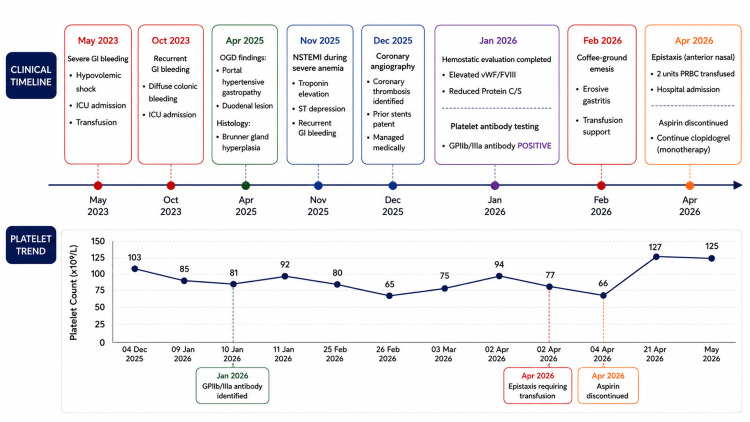
Platelet trend and major clinical events during the patient’s clinical course This figure illustrates the platelet trend and major clinical events, including major bleeding episodes, PRBC transfusions, antiplatelet therapy modifications, cardiac events, and diagnostic milestones during the patient’s clinical course. The upper panel depicts major bleeding, thrombotic, diagnostic, and therapeutic milestones. The lower panel demonstrates serial platelet counts from December 2025 to May 2026, showing persistent thrombocytopenia followed by hematologic stabilization after modification of antiplatelet therapy. NSTEMI: Non-ST-elevation myocardial infarction; LAD: Left anterior descending artery; LCX: Left circumflex artery; PRBC: Packed red blood cells. Source: This figure was created by the authors using Canva (Canva Pty Ltd., Sydney, Australia).

Although the platelet antibody result became available earlier during the clinical course, it was not initially integrated into management. The patient therefore continued to experience recurrent bleeding with ongoing cycles of antiplatelet withdrawal and reintroduction. During a subsequent admission, the previously obtained platelet antibody result was revisited in the context of the patient’s longitudinal trajectory. Recognition of the positive platelet antibody result together with the overall longitudinal clinical pattern prompted reconsideration of a possible platelet-mediated contribution to the bleeding phenotype, leading to permanent discontinuation of aspirin while continuing clopidogrel because of ongoing cardiovascular risk. Longitudinal platelet trend and major clinical events during the patient’s clinical course are shown in Figure 2.

Following this intervention, the patient demonstrated cessation of overt bleeding, elimination of transfusion requirements, and sustained improvement in platelet counts over subsequent weeks. At follow-up more than two months after permanent aspirin withdrawal, the patient remained free of overt bleeding, with stable hemoglobin levels and sustained platelet recovery to approximately 125 × 10⁹/L, without further transfusion requirement. This represented the longest sustained period of hematologic stability observed during the documented clinical course. Although a causal relationship cannot be established from a single case, the temporal association between aspirin discontinuation, platelet recovery, cessation of bleeding, and elimination of transfusion requirements was consistent with the proposed hypothesis of platelet-mediated hemostatic instability.

## Discussion

This case suggests that the patient’s prolonged clinical instability was not explained by structural gastrointestinal pathology alone but may have reflected platelet-mediated hemostatic vulnerability operating within the unstable physiological background of chronic liver disease and repeatedly destabilized by antiplatelet therapy. The observed clinical course supports the hypothesis that impaired primary hemostatic resilience, when perturbed by antiplatelet exposure, contributed to a recurrent cycle of bleeding, anemia, cardiac stress, and therapeutic re-escalation.

Contemporary understanding of hemostasis in cirrhosis has evolved from the traditional concept of a hypocoagulable state toward a model of rebalanced hemostasis. However, this terminology risks implying physiologic stability. Rather than a stable balance, the cirrhotic hemostatic system is better conceptualized as a metastable equilibrium that is highly sensitive to perturbation. In this state, baseline compensation may be sufficient for temporary stability but inadequate to withstand additional physiologic or pharmacologic stress. This is particularly relevant during acute illness, bleeding, anemia, infection, renal dysfunction, or exposure to antiplatelet therapy, all of which may destabilize the hemostatic balance in cirrhosis [10].

In this patient, multiple structural lesions - including hemorrhoidal disease, portal hypertensive gastropathy, erosive gastritis, and benign duodenal abnormalities - were repeatedly identified but failed to explain the chronicity, severity, and transfusion dependence of bleeding. These lesions are better understood as permissive substrates rather than primary drivers. The persistence of severe bleeding despite endoscopic interventions indicates that structural pathology alone was insufficient to account for the observed phenotype. Instead, the clinical course reflects failure of effective primary hemostasis superimposed on structurally vulnerable mucosa.

The case therefore challenges the common assumption that once a structural lesion is identified in a cirrhotic patient, the mechanism of bleeding has been sufficiently explained. In metastable systems, apparent short-term equilibrium should not be interpreted as physiologic robustness. Rather, compensatory reserve may be sufficient to maintain temporary clinical quiescence but fail abruptly under additional perturbation. In cirrhosis, bleeding, anemia, infection, inflammation, renal dysfunction, and antiplatelet exposure may collectively reduce hemostatic reserve and unmask latent instability. This framework better explains why some patients experience prolonged periods of relative stability punctuated by abrupt episodes of severe hemorrhage disproportionate to the structural findings.

Platelet glycoprotein IIb/IIIa dysfunction: final common pathway collapse of primary hemostasis

The most mechanistically relevant finding in this case was the presence of platelet glycoprotein IIb/IIIa antibody reactivity. Integrin αIIbβ3, or GPIIb/IIIa, represents the final common pathway of platelet aggregation. Platelet adhesion and activation are mediated by upstream pathways involving glycoprotein Ib-vWF interaction, ADP-P2Y12 signaling, thromboxane A2 amplification, intracellular calcium flux, and talin- and kindlin-mediated inside-out signaling. These pathways converge on conformational activation of αIIbβ3, enabling fibrinogen binding and inter-platelet cross-linking.

This process enables fibrinogen-mediated bridging between adjacent platelets and stabilization of the primary hemostatic plug. Failure of integrin αIIbβ3 activation therefore results in defective fibrinogen bridging and unstable clot formation, particularly under conditions of high shear stress, rendering mucosal and microvascular beds especially vulnerable to persistent bleeding. In these vascular territories, bleeding depends less on deficiencies in secondary coagulation and more on defective formation and maintenance of the primary platelet plug.

This represents not merely a peripheral platelet abnormality but a final common pathway failure in which upstream signaling may remain partially intact but cannot translate into stable clot architecture. In this context, upstream preservation of platelet adhesion does not compensate for downstream aggregation failure, resulting in formation of transient platelet plugs that are mechanically unstable and prone to disruption under physiologic shear conditions. The clinical phenotype suggests not complete absence of hemostatic function but impaired hemostatic resilience, in which baseline compensation was sufficient for temporary stability yet inadequate to withstand additional pharmacologic or physiologic stress.

In this patient, antibody reactivity against GPIIb/IIIa provides a biologically plausible explanation for persistent bleeding despite relatively preserved conventional coagulation parameters. Importantly, this phenotype does not conform to classical chronic immune thrombocytopenia. Rather, it reflects a qualitative platelet dysfunction superimposed on quantitative thrombocytopenia within the broader context of hepatic and endothelial dysregulation.

Although the presence of glycoprotein IIb/IIIa antibody reactivity raises the possibility of an immune-mediated platelet disorder, the overall clinical phenotype in this case does not support classical chronic immune thrombocytopenia as the primary mechanism. Immune thrombocytopenia is typically characterized by thrombocytopenia that is not consistently linked to pharmacologic triggers and often demonstrates a more unpredictable clinical course. In contrast, this patient exhibited a reproducible pattern in which bleeding severity and platelet trends were temporally associated with aspirin exposure and withdrawal. Furthermore, the degree of bleeding was disproportionate to the level of thrombocytopenia, indicating a dominant qualitative platelet dysfunction rather than isolated immune-mediated platelet destruction. Taken together, these features suggest that while immune-mediated mechanisms may contribute to platelet abnormalities, they do not adequately explain the observed clinical trajectory and are unlikely to represent the principal driver of disease in this case. Moreover, stable platelet recovery following aspirin withdrawal alone, without corticosteroids, intravenous immunoglobulin, thrombopoietin receptor agonists, or other immune-directed therapy, would be atypical for untreated chronic immune thrombocytopenia.

Emerging mechanistic literature suggests that antiplatelet antibodies may influence platelet clearance, receptor biology, desialylation, and apoptosis, supporting the possibility that antibody positivity may be biologically meaningful even when the clinical phenotype does not fit classical immune thrombocytopenia [11]. Furthermore, durable hematologic stabilization after aspirin withdrawal without corticosteroids, intravenous immunoglobulin, thrombopoietin receptor agonists, or other ITP-directed therapy argues against untreated chronic immune thrombocytopenia as the principal mechanism. Instead, the observed behavior supports interruption of a platelet-mediated destabilization process superimposed upon chronic liver disease-related thrombocytopenia.

However, the platelet antibody result should be interpreted cautiously. Platelet antibody assays have recognized limitations, including variable sensitivity and specificity, lack of complete assay standardization, and inability to distinguish pathogenic antibodies from incidental immune reactivity in all cases. In addition, functional platelet aggregation studies were not available in this patient; therefore, direct confirmation of antibody-mediated platelet dysfunction could not be established. The antibody result is therefore best interpreted as supportive of the proposed platelet-mediated hypothesis when considered alongside the longitudinal clinical pattern rather than as definitive proof of causality.

The liver-platelet-endothelium axis: loss of hemostatic buffering capacity

The hemostatic phenotype in this case cannot be understood without considering the integrated liver-platelet-endothelium axis. Chronic liver disease contributes to thrombocytopenia through reduced thrombopoietin synthesis and splenic sequestration. Simultaneously, endothelial activation leads to increased circulating vWF and altered ADAMTS13 activity, reflecting a compensatory but dysregulated attempt to preserve primary hemostasis.

At a molecular level, vWF facilitates platelet adhesion and partially compensates for reduced platelet number. However, effective hemostasis requires not only adhesion but also aggregation and clot consolidation. In the presence of impaired αIIbβ3-mediated aggregation, elevated vWF cannot restore stable clot formation. This creates a paradoxical state in which laboratory markers suggest compensatory activation, yet clinically significant bleeding persists. This dissociation between laboratory compensation and clinical bleeding underscores that effective hemostasis depends on coordinated function across multiple physiologic levels rather than isolated parameter normalization.

The VWF/ADAMTS13 axis is increasingly recognized as a clinically relevant part of liver-related hemostatic dysregulation. VWF/ADAMTS13 imbalance has been associated with bleeding and adverse outcomes in acute liver failure, emphasizing that endothelial and primary-hemostatic abnormalities may be more clinically informative than global coagulation tests alone in selected liver disease states [12]. In advanced chronic liver disease, altered vWF processing has also been linked with portal hypertension and clinical outcomes, supporting the view that elevated vWF is not simply a protective compensatory marker but part of a complex endothelial-hemostatic phenotype [13]. Reduced ADAMTS13 activity in advanced liver disease may permit persistence of ultra-large von Willebrand multimers, increasing endothelial-platelet interaction and contributing to a paradoxical state in which thrombotic signaling and hemorrhagic vulnerability coexist. However, when downstream platelet aggregation is impaired, increased adhesive signaling alone cannot ensure durable clot integrity.

The observed reduction in Protein C and Protein S levels further reflects impaired hepatic synthetic function and erosion of endogenous anticoagulant pathways. Rather than conferring a straightforward prothrombotic state, these abnormalities more likely indicate loss of hemostatic buffering capacity, rendering the system increasingly susceptible to directional shifts toward either bleeding or thrombosis depending on external stressors.

Conventional coagulation assays such as PT and INR fail to capture these dynamics. These tests primarily assess fibrin generation under static laboratory conditions and do not reflect platelet adhesion, platelet aggregation, endothelial interaction, or stability of primary hemostatic plug formation. In this patient, reliance upon such parameters likely contributed to under-recognition of a profound defect in primary hemostasis.

Anemia as a central hemodynamic driver of cardiac stress

A critical feature of this case is that anemia functioned not merely as a consequence of bleeding but as an active driver of downstream pathology. Reduction in hemoglobin decreases arterial oxygen content, triggering compensatory sympathetic activation, tachycardia, increased stroke volume, and elevated cardiac output. These responses increase myocardial oxygen demand while simultaneously reducing oxygen delivery, thereby creating a state of supply-demand mismatch.

This mechanism underlies type 2 myocardial infarction and explains recurrent troponin elevation in the absence of primary coronary plaque instability. The subendocardial myocardium is particularly vulnerable to this imbalance, and repeated episodes of anemia can generate ischemic signals that are clinically difficult to distinguish from acute coronary syndromes. Recurrent troponin elevation in this setting may therefore represent a biologically understandable downstream consequence of hemorrhagic instability rather than independent primary cardiovascular destabilization. Failure to recognize this distinction risks therapeutic escalation that inadvertently worsens the initiating process.

Beyond acute mismatch, chronic anemia has broader cardiovascular implications. Sustained neurohormonal activation, increased cardiac workload, impaired myocardial energetics, reduced exercise tolerance, and progressive ventricular remodeling may collectively contribute to cumulative cardiovascular vulnerability. Contemporary heart failure literature recognizes anemia and iron deficiency as clinically important contributors to adverse symptoms and outcomes, and contemporary heart failure guidelines further reinforce the importance of identifying and treating these contributors in appropriate patients [14].

In this patient, recurrent severe anemia likely did more than provoke episodic myocardial injury; it repeatedly reinforced cardiology involvement and recurrent escalation of antiplatelet therapy, thereby perpetuating the broader instability cycle. Each major bleeding episode therefore did not terminate with transfusion alone but generated a recurrent physiologic cardiac stress state that repeatedly re-legitimized escalation of antithrombotic therapy.

Aspirin as a system destabilizer and clopidogrel tolerance: a mechanistic and pharmacogenomic perspective

A key observation in this case is the differential clinical impact of aspirin and clopidogrel. Aspirin irreversibly inhibits platelet cyclooxygenase-1, suppressing thromboxane A2 synthesis and eliminating a major amplification pathway in platelet activation. In a system already compromised by thrombocytopenia and impaired αIIbβ3-mediated aggregation, loss of thromboxane-driven recruitment may disproportionately collapse residual platelet functional reserve.

In this context, aspirin likely eliminated a critical remaining amplification reserve within an already compromised platelet signaling network, thereby converting compensated dysfunction into clinically overt hemostatic collapse. Additionally, aspirin exerts direct gastrointestinal mucosal toxicity independent of platelet inhibition, further amplifying bleeding risk. The combination of systemic platelet suppression and local mucosal injury likely magnified hemorrhagic vulnerability in this patient. Literature comparing aspirin, clopidogrel, and dual antiplatelet therapy supports the clinical relevance of drug-specific gastrointestinal injury patterns rather than treating all antiplatelet exposure as equivalent [15].

Clopidogrel, in contrast, inhibits ADP-mediated P2Y12 signaling. Although this pathway is also central to platelet activation, its physiologic impact depends partly upon hepatic bioactivation and interindividual pharmacogenomic variability. Clopidogrel requires CYP2C19-mediated conversion to its active metabolite, and reduced-function polymorphisms may attenuate antiplatelet activity. Although pharmacogenomic testing was not performed in this patient, reduced clopidogrel activation could plausibly contribute to its relative tolerability compared with aspirin. The CPIC guideline for CYP2C19 and clopidogrel supports the broader concept that CYP2C19 genotype materially influences clopidogrel activation and clinical antiplatelet effect [16].

The reproducible dechallenge-rechallenge pattern observed with aspirin represents one of the most compelling clinical observations in this case. While this finding cannot independently establish the entire proposed biological mechanism, it provides strong support for aspirin as a major contributor to the recurrent bleeding phenotype and is consistent with the proposed platelet-mediated hemostatic instability framework. The differential biologic response observed in this patient highlights how clinically apparent drug sensitivity patterns may reflect underlying heterogeneity in platelet signaling architecture, receptor expression, mucosal susceptibility, or antiplatelet drug metabolism, even in the absence of formal genomic characterization.

Platelet-mediated hemostatic instability cycle: a reproducible clinicophysiologic pattern

We describe a reproducible clinicophysiologic pattern, described here as a platelet-mediated hemostatic instability cycle, in which interventions targeting thrombotic risk exacerbate bleeding, whereas ​​​​​interventions targeting bleeding increase perceived thrombotic risk, resulting in recurrent clinical instability.

In this patient, the sequence was repeatedly observed: bleeding led to anemia; anemia led to cardiac stress and troponin elevation; troponin elevation triggered escalation of antiplatelet therapy; and antiplatelet exposure precipitated further bleeding.

This was not proposed as a confirmed disease mechanism but as a hypothesis-generating clinicophysiologic framework derived from a reproducible longitudinal pattern observed across multiple admissions, with improvement after aspirin was permanently withdrawn following recognition of a possible platelet-mediated contribution. While each individual component of this cycle is well described in prior literature, their integration into a self-sustaining feedback system remains underrecognized in clinical practice. Importantly, the cycle observed in this patient should not be interpreted as a purely therapeutic artifact but as a biologically amplified feedback system operating across multiple physiologic domains. Hemorrhage generated anemia; anemia generated myocardial stress; myocardial stress increased perceived thrombotic vulnerability; and antiplatelet intensification reduced residual hemostatic reserve, thereby precipitating recurrent hemorrhage. The clinical significance of this framework lies in recognizing that repeated therapeutic escalation may unintentionally reinforce the initiating pathology when mechanistic integration is absent.

The proposed terminology is therefore intended as a descriptive clinicophysiologic framework derived from the observed longitudinal behavior in this patient rather than as a newly defined disease entity.

Comparison with existing literature

Similar clinical dilemmas have been described in patients with acute coronary syndrome or prior percutaneous coronary intervention who subsequently develop major gastrointestinal bleeding during antiplatelet therapy. These reports and guidelines emphasize the difficulty of balancing ischemic protection against hemorrhagic risk and support individualized modification of antithrombotic therapy in high-risk gastrointestinal bleeding contexts [17].

Antiplatelet therapy has also been associated with bleeding and decompensation events in patients with cirrhosis, further supporting the clinical relevance of antiplatelet exposure as a destabilizing factor in liver disease populations [18]. However, most available reports focus primarily on antithrombotic strategy or procedural decision-making rather than on an integrated platelet-level mechanism driving recurrent instability.

In contrast, the present case incorporates several interacting mechanistic layers simultaneously: chronic liver disease with metastable hemostasis, glycoprotein IIb/IIIa antibody-associated qualitative platelet dysfunction, endothelial dysregulation, recurrent anemia-mediated myocardial stress, and repeated antiplatelet re-escalation. The novelty of this case therefore does not lie in the coexistence of bleeding and thrombosis alone but in the description of a reproducible clinical pattern consistent with a platelet-mediated hemostatic instability cycle in which these processes interacted longitudinally as a self-sustaining physiologic loop.

Importantly, interruption of this cycle following permanent aspirin withdrawal resulted in the longest sustained period of hematologic stability observed during the patient’s documented clinical course, further supporting the clinical plausibility of the proposed framework.

To our knowledge, reports describing glycoprotein IIb/IIIa antibody-associated platelet dysfunction in chronic liver disease are exceedingly limited, and we were unable to identify published cases demonstrating a reproducible longitudinal bleeding-anemia-cardiac stress-antiplatelet feedback cycle analogous to that observed here. Although antiplatelet-associated bleeding in cirrhosis and qualitative platelet dysfunction have been independently described, their integration into a unified platelet-mediated instability framework appears to be underrepresented in the literature.

Counterargument: can structural portal hypertensive disease alone explain the course?

An alternative explanation is that bleeding was primarily driven by portal hypertensive mucosal disease, with platelet abnormalities representing secondary epiphenomena of cirrhosis. However, this interpretation is not supported by the temporal behavior observed throughout the clinical course.

Structural lesions remained relatively stable, whereas bleeding severity fluctuated in direct relation to aspirin exposure. Bleeding consistently improved following aspirin withdrawal and recurred after reintroduction despite persistence of underlying mucosal pathology. Structural lesions do not typically exhibit such tightly drug-linked oscillatory behavior in the absence of a deeper hemostatic driver.

The temporal dissociation between stable anatomical findings and dynamic clinical severity therefore supports a clinically important platelet-mediated mechanism. If structural pathology alone were sufficient to explain the clinical course, such reproducible pharmacologic modulation of bleeding would not be expected.

Precision medicine perspective: mechanism-based interpretation in the genomic era

Although this case was not grounded in formal genomic testing, it aligns closely with contemporary precision medicine principles. Platelet biology is increasingly understood to be influenced by interindividual variability in receptor signaling, immune modulation, platelet reactivity, and drug metabolism. Variants affecting COX-1 activity, P2Y12 signaling, integrin expression, and CYP2C19-mediated drug activation may all contribute to differences in bleeding and thrombotic phenotypes.

The differential response to aspirin and clopidogrel observed in this patient may plausibly reflect such variability. While definitive pharmacogenomic confirmation is unavailable, the clinical phenotype supports a mechanism-based interpretation rather than a purely empirical one. This reinforces the importance of individualized therapeutic strategies guided by functional biologic behavior rather than protocol-driven algorithms alone.

Diagnostic integration failure as a system-level problem

Perhaps the most instructive aspect of this case is that the critical diagnostic clue was not absent; rather, it was generated but not synthesized. The platelet antibody result existed before it altered management. The delay therefore reflected a gap in integrative clinical reasoning rather than a failure of investigation.

The patient’s clinical course was repeatedly interpreted through isolated specialty-specific lenses rather than through integrated longitudinal physiology. Persistent anchoring to structural gastrointestinal pathology, combined with fragmentation of care across specialties, delayed recognition of a unifying platelet-mediated mechanism.

This case exemplifies how specialty-specific silos and overreliance on conventional coagulation parameters may obscure broader physiologic patterns in complex multisystem disease.

Synthesis and clinical implication

In this patient, qualitative platelet dysfunction, thrombocytopenia, endothelial dysregulation, recurrent anemia, and pharmacologic platelet inhibition interacted to produce sustained hemostatic instability.

The resulting anemia acted as a physiologic amplifier, repeatedly triggering cardiac stress and therapeutic escalation, thereby perpetuating a closed-loop cycle of clinical deterioration. Recognition of this proposed cycle and modification of antiplatelet therapy were followed by the longest sustained period of hematologic stability observed during the patient’s documented clinical course and underscore the importance of mechanism-based clinical reasoning in complex bleeding-thrombosis syndromes.

This case illustrates that severe recurrent bleeding in chronic liver disease may arise from dynamic failure of primary hemostasis rather than structural pathology alone. In this patient, qualitative platelet dysfunction, thrombocytopenia, endothelial dysregulation, recurrent anemia, and pharmacologic platelet inhibition appeared to interact to produce sustained hemostatic instability. Recognition of this proposed cycle and modification of antiplatelet therapy were followed by the longest sustained period of hematologic stability observed during the documented clinical course. These observations support a hypothesis-generating framework and underscore the importance of mechanism-based clinical reasoning in complex bleeding-thrombosis syndromes.

Learning points

Disproportionate bleeding in cirrhosis should prompt evaluation of platelet function, even in the presence of identifiable structural lesions.

Platelet glycoprotein IIb/IIIa antibody reactivity may support investigation of qualitative platelet dysfunction in selected patients with disproportionate bleeding.

Severe anemia may function as an active driver of myocardial stress and recurrent cardiovascular escalation rather than merely a consequence of bleeding.

Aspirin may act as a major destabilizer in patients with underlying platelet functional vulnerability.

A platelet-mediated hemostatic instability cycle may be a useful hypothesis-generating framework when bleeding, anemia, cardiac stress, and antiplatelet escalation repeatedly reinforce one another.

## Conclusions

This case supports the hypothesis that recurrent bleeding in chronic liver disease may arise from platelet-mediated failure of primary hemostasis in addition to structural gastrointestinal pathology. In this patient, glycoprotein IIb/IIIa antibody reactivity, chronic thrombocytopenia, endothelial dysregulation, aspirin exposure, and anemia-driven cardiac stress appeared to interact to produce a reproducible bleeding-cardiac-antiplatelet cycle. The sustained clinical stabilization observed after aspirin withdrawal was consistent with interruption of the proposed platelet-mediated hemostatic instability cycle and supports the clinical plausibility of this hypothesis.

Beyond the individual patient, this case emphasizes the importance of longitudinal pattern recognition, platelet-focused evaluation, and integrated mechanism-based clinical reasoning when conventional structural and coagulation-based explanations fail to account for the observed severity of bleeding. Earlier recognition of platelet-mediated hemostatic vulnerability may permit timely modification of antiplatelet strategies, reduce transfusion dependence, prevent recurrent hospitalization, and avoid therapeutic decisions that unintentionally perpetuate clinical deterioration. Although derived from a single case, the proposed platelet-mediated hemostatic instability cycle should be regarded as a hypothesis-generating clinical framework that warrants validation in future studies.
